# *Aspergillus niger*-fermented watermelon rind enhances growth, immunity, and heat stress resilience in Asian seabass (***Lates calcarifer***)

**DOI:** 10.1016/j.vas.2026.100871

**Published:** 2026-09-14

**Authors:** Ehsan Ahmadifar, Ali Arshadi, Sedigheh Mohammadzadeh, Majid Khanzadeh, Mostafa Khajeh, Amin Oujifard, Mohsen Shahriari Moghadam, Mohsen Abdel-Tawwab

**Affiliations:** a Department of Fisheries, Faculty of Natural Resources, University of Zabol, Zabol, Iran; b Department of Fisheries, Faculty of Nano and Bio Science and Technology, Persian Gulf University, Bushehr 7516913817, Iran; c Faculty of Veterinary Medicine, Amol University of Special Modern Technologies, Amol, Iran; d Animal Biological Product Research Group, Academic Center for Education, Culture and Research (ACECR), Tehran Organization, 1314744513, Tehran, Iran; e Department of Chemistry, University of Zabol, Zabol, Iran; f Departement of Environmental Sciences, Faculty of Natural Resources, University of Zabol, Zabol, Iran; g Department of Fish Biology and Ecology, Central Laboratory for Aquaculture Research, Agricultural Research Center, Abbassa, Abo-Hammad, Sharqia 44662, Egypt

**Keywords:** Aspergillus niger, Fermentation, Watermelon, Hyperthermia, Asian seabass

## Abstract

An 8-week feeding study was undertaken to assess the impacts of incorporating yeast-fermented watermelon rind powder (FWRP), produced using *Aspergillus niger*, on growth performance, antioxidant capacity, immune responses, and thermal stress tolerance in Asian seabass fingerlings. Three hundred fish, averaging 5.68 ± 0.32 g in initial weight, were assigned at random to 12 tanks, with 25 individuals stocked in each tank. The fish received the assigned diets with different FWRP levels of 0.0, 10, 20, and 30 g/kg feed (FWRP0, FWRP10, FWRP20, and FWRP30, respectively) over the course of 8 weeks. At the end of the feeding trial, fish were exposed to thermal stress by increasing water temperature from 23 to 32 °C at 3 °C day⁻¹ and maintaining it at 32 °C for 3 days. The findings showed that the dietary FWRP notably stimulated growth, antioxidant, and serum immune parameters of Asian seabass fingerlings relative to the control group (*P* < 0.05). Fish fed on 30 g FWRP/kg feed produced the highest values of growth, digestive enzymes, antioxidant, and immune indices. Activities of alkaline phosphatase and aspartate aminotransferase declined as levels of FWRP in diets increased, and their lowest levels were detected in fish fed on FWRP20 and FWRP30. The findings revealed that the use of fermented FWRP may serve as an effective feed additive for Asian seabass, promoting growth performance, enhancing antioxidant and immune responses, and increasing resistance to hyperthermia stress.

## Highlights

• FWRP boosted growth and health in Asian seabass.
• 30 g/kg FWRP optimized digestive and immune function.
• Dietary FWRP lowered stress-related enzyme activity.
• Fermented rind enhanced resistance to hyperthermia.

## 1 Introduction

Asian seabass (*Lates calcarifer*) is widely regarded as an excellent species for aquaculture diversification, owing to its high adaptability to varying environmental conditions, strong biological performance, and considerable market demand (Yue, 2025). During the last two decades, *L. calcarifer* production has grown significantly, primarily due to the expansion and intensification of aquaculture operations in leading producing regions to meet increasing consumer demand (Reyshari et al., 2019; Szucs et al., 2018; Yue, 2025). However, higher stocking densities can impose stress on fish by deteriorating water quality and intensifying social interactions and hierarchical competition within the population. These conditions may elevate oxidative stress, compromise immune function, and increase susceptibility to infectious diseases.

Global warming, in particular, has become a major global concern, as it significantly influences the behavior, physiological processes, and overall health of organisms, thereby posing a serious challenge to sustainable aquaculture production (Islam et al., 2022; Mugwanya et al., 2022). Recent projections suggest that water temperatures may increase by approximately 2–4 °C over the next 80 years. Furthermore, the Cheung model indicates that even a 1 °C rise in global temperature could lead to a reduction of >3 million metric tons in global aquaculture production (Cheung et al., 2016; Singh & Sarma, 2025). Temperature fluctuations can have particularly detrimental effects on the fish immune system, as thermal stress may directly lead to mortality or weaken disease resistance, thereby increasing susceptibility to pathogen invasion (Abisha et al., 2022; Islam et al., 2022; Mugwanya et al., 2022).

Mitigating the adverse impacts of heat stress through appropriate feeding strategies is essential. In this context, the incorporation of functional feed additives into fish diets has been demonstrated to promote growth performance, enhance nutrient utilization, strengthen immune responses, and increase resilience to environmental stressors in aquaculture species (Tadese et al., 2022). Exposure to heat stress has been shown to be alleviated by various dietary supplements. For instance, oregano essential oil, *Hericium erinaceus* powder, and *Yucca schidigera* have been reported to improve stress tolerance in *Oreochromis niloticus* (Magouz et al., 2022). Similarly, the probiotic *Clostridium butyricum* enhanced thermal resilience in kuruma shrimp (*Marsupenaeus japonicus*) (Duan et al., 2017), whereas mannan oligosaccharides and allicin were found to strengthen stress resistance in gilthead seabream (*Sparus aurata*) (Khosravi-Katuli et al., 2021) and striped catfish (*Pangasianodon hypophthalmus*) (Mahmoud et al., 2023), respectively. These improvements are primarily linked to the modulation of antioxidant systems and immune function.

Watermelon rind powder (WRP) has recently attracted attention as a promising feed additive due to its nutrient-rich composition, including minerals, lipids, carbohydrates, vitamins, proteins, phytochemicals, and citrulline (Mendes et al., 2026). Among these components, carbohydrates constitute the predominant fraction and are an important source for pectin extraction (Méndez et al., 2021). Pectin, in turn, has been recognized as a novel prebiotic with diverse biological functions (Blanco-Perez et al., 2021; Naqash et al., 2017).

Notably, WRP has been reported to exhibit strong antioxidant capacity, characterized by a high ability to scavenge free radicals. This activity is largely attributed to its rich content of bioactive compounds, including l-citrulline, phenolic constituents—particularly 4-hydroxybenzoic acid and vanillin—as well as saponins, terpenoids, phenols, and alkaloids (Kumar et al., 2018; Mendes et al., 2026; Tarazona-Díaz et al., 2013). In this context, Van Doan et al. (2020) evaluated the impacts of dietary WRP on immune responses, growth performance, and disease resistance in *O. niloticus*. Their findings demonstrated significant improvements in growth indices, as well as enhanced skin mucus and serum immune parameters, alongside increased resistance to *Streptococcus agalactiae* infection. The most pronounced benefits were observed at a dietary inclusion level of 40 g/kg. Furthermore, Van Doan et al. (2021) reported that the combined application of WRP and the probiotic *Lactobacillus plantarum* further enhanced growth, immune function, and resistance to *S. agalactiae*. Fermentation of WRP using yeast (FWRP) has been shown to further enhance its bioactive properties compared to the unfermented form. Supporting this, Khajeh et al. (2026) demonstrated that FWRP contained significantly higher levels of total phenolics and flavonoids than unfermented WRP, resulting in superior antioxidant activity.

In light of these promising characteristics, this research was undertaken to investigate the potential of yeast-fermented FWRP as a dietary supplement for enhancing growth performance, immune and antioxidant functions, and resistance to heat stress in Asian seabass fingerlings.

## 2 Methods

### 2.1 Dietary treatments

Fresh WRP was thoroughly washed with water and dried using an oven set at 40 °C. The oven-dried samples were then ground into a uniform powder. To remove possible contaminants, the watermelon peel powder was sterilized by autoclaving. Solid-State Fermentation was performed using *Aspergillus niger*. A fungal suspension with a concentration of 1 × 10⁸ colony-forming units (CFU) per g of substrate was added to the watermelon peel powder. The substrate humidity was adjusted to 60%, and the samples were held under incubation conditions at 30 °C for 96 h. During the fermentation process, the fungus produced extracellular enzymes, including cellulases and proteases, to degrade complex carbohydrate and protein compounds and boost the bioactive constituents and bioavailability of nutrients. After the fermentation period, the samples were dried at 50 °C to stop microbial activity. Finally, the dried product was ground again and stored as fermented watermelon rind powder for use in experimental diets. The proximate composition of FWRP was 9.3%, 14.0%, 2.2%, 6.6%, 8.3%, and 61.0% for moisture, protein, lipids, ash, fiber, and total carbohydrates, respectively.

The test diets (Table 1) were mixed and supplemented with varying concentrations of FWRP, including 0, 10, 20, and 30 g/kg feed, represented by FWRP0 (the control), FWRP10, FWRP20, and FWRP30, respectively. For diet preparation, all dry ingredients were thoroughly blended for 15 min. Fish oil was then introduced and blended for an additional 10 min, followed by the incorporation of water and further mixing for another 10 min until a uniform dough was obtained. The resulting dough was subsequently extruded to form the final feed pellets (2 mm). The pellets were placed in an oven and dried at 45 °C until the moisture level fell below 10%. Once dried, the diets were sealed in containers and kept at −20 °C until required for use.

**Table 1:** Nutrient composition and ingredient profile of experimental diets formulated with varying levels of fermented watermelon rind (FWRP) on a dry matter basis.

Ingredients a (g/kg)	FWRP0	FWRP10	FWRP20	FWRP30
Fish meal b	600	600	600	600
Soybean meal c	80	80	80	80
Wheat gluten b	80	80	80	80
Beef gelatin b	20	20	20	20
Wheat middling	45	45	45	45
Fish oil b	90	90	90	90
Soy lecithin c	40	40	40	40
Vitamin premix d	7	7	7	7
Mineral premix e	7	7	7	7
Antioxidant f	1	1	1	1
Corn meal b	30	20	10	0
FWRP	0	10	20	30
Totals	1000	1000	1000	1000
Proximate analysis				
Chemical analysis (g/100 g)				
Moisture	5.60	5.68	5.72	5.76
Crude protein	56.31	56.24	56.29	56.34
Crude lipids	16.92	16.86	16.82	16.75
Ash	9.00	9.03	9.07	9.11
Crude fiber	2.93	2.98	3.04	3.09
Nitrogen-free extract g	9.24	9.21	9.06	8.95
Gross energy (MJ/g) h	21.56	21.51	21.48	21.45

a Ingredients are expressed on a dry-weight basis (g/kg): gluten (714 g/kg crude protein (cp), 41 g/kg crude lipid (CL); soybean meal (410 g/kg CP, 42 g/kg CL); fish meal (735 g/kg CP, 107 g/kg CL); wheat beef gelatin (850 g/kg CP, 42 g/kg CL); wheat middlings (120 g/kg CP, 30 g/kg CL). Corn meal (71 g/kg CP, 20 g/kg CL).

b Supplied by Beyza Feed Mill, Shiraz, Iran.

c Provided by Behpak Industrial Company, Behshahr, Mazandaran, Iran.

d Butylated hydroxytoluene (BHT), Garmab Shimi, Iran.

e Vitamin premix (per kg): 180,000 mg; antioxidant 500 mg; vitamins: B12 60,000 mg; K3 1500 mg, A 5000,000 IU; E 3000 mg; D3 500,000 IU; folic acid 3000 mg; B1 6000 mg; B2 24,000 mg; B5 (pantothenic acid) 52,000 mg; B6 18,000 mg; nicotinamide carrier up to 1 kg (Damloran Pharmaceutical Company, Borujerd, Iran).

f Mineral premix (per kg): iron 10,000 mg; manganese 20,000 mg; zinc 15,000 mg; copper 3000 mg; potassium iodate 300 mg; carrier up to 1 kg (Microvit®, Razak Laboratories, Tehran, Iran).

g Nitrogen-free extract (NFE) = 100 − (crude protein + crude fat + crude fiber + ash + moisture).

h Gross energy (MJ kg⁻¹) = (crude protein × 23.6) + (crude fat × 39.5) + (NFE × 17.2).

### 2.2 Experimental condition

The experiment was carried out in line with ethical standards and responsible animal care procedures and protocols approved by the Faculty of Sciences, University of Tehran, Iran (Approval No. 357; 8 November 2000). For this study, Asian seabass, *L. calcarifer*, juveniles were obtained from the Aquatic Research Laboratory, Persian Gulf University (Bushehr, Iran) and then transferred to the research hall of the center. The fish (5.68 ± 0.32 g) were kept in two 500-L tanks for 2 weeks to acclimatize to environmental conditions and fed with a basal diet 3 times a day. During the acclimatization period, commercial feed from Faradaneh, Shahrekord, Iran, was used. Upon completion of the acclimation period, 300 *L. calcarifer* were individually transferred and distributed into twelve 300-L tanks and allocated to 4 experimental treatments with 3 replicates (i.e., 25 *L. calcarifer* per tank). The fish were given the respective dietary treatments for eight weeks. Fish feeding was done in thrice daily meals (9:00, 12:00, and 17:00 h) to visual satiation. Fish were provided with feed at a daily ration equivalent to 3% of the total biomass in each tank throughout the 8-week experimental period. The feeding ration was adjusted every 10 days according to changes in the biomass of fish in each tank. The test tanks were filled with 200 L of seawater that had been filtered through sand and treated with UV. Water quality parameters were maintained at 39.1 ± 0.6‰ salinity, 23.5 ± 0.5 °C temperature, 5.8 ± 0.2 mg/L dissolved oxygen, and pH 7.9 ± 0.2, under a natural photoperiod (12L/12D). At the termination of the trial, all fish were not fed for 24 h before harvesting for final measurements, and the final body weights were recorded. Feed conversion ratio (FCR), weight gain (WG), survival rate (SR), feed intake (FI), and specific growth rate (SGR) were measured using standard formulae (Carneiro et al., 2020).

### 2.3 Sampling

Upon completion of the 8-week feeding experiment, fish underwent a 24-h fasting period before sampling. From each tank, 3 fish (i.e., 3 fish per replicate, totaling 9 *L. calcarifer* per treatment) were randomly chosen and anesthetized with 2-phenoxyethanol (0.5 mL/L; Aalamifar et al., 2020). Blood samples were obtained from the caudal peduncle of the fish using sterile 1-mL syringes and subsequently centrifuged at 3000 × g for 15 min under ambient temperature conditions to facilitate serum separation. The resulting serum samples were carefully divided into aliquots and preserved at −80 °C until further biochemical assessments (Khanzadeh et al., 2025). Following blood collection, the fish were dissected, and liver and intestinal tissues were excised from the same individuals for subsequent analyses. Intestinal samples were processed to determine digestive enzyme activities, whereas liver samples were used to assess hepatic enzyme profiles. All tissue specimens were preserved at −80 °C pending further analysis.

### 2.4 Intestinal digestive and liver enzymes

Intestinal tissues were homogenized in ice-cold buffer, and the resulting homogenates were centrifuged to collect the supernatant for analysis of digestive enzyme activities. Amylase, protease, and lipase activities were quantified following the methods outlined by Aalamifar et al. (2020). Specific enzyme activities were calculated by normalizing to the protein content of each sample and expressed as enzyme units per mg of protein.

Liver samples were processed in a similar manner under cold conditions, and the supernatants obtained after centrifugation were used to evaluate hepatic enzyme activities. Hepatic marker enzymes, including alanine aminotransferase (ALT), aspartate aminotransferase (AST), and alkaline phosphatase (ALP), were measured using commercial assay kits (Pars Azmun, Iran) in accordance with the manufacturer’s guidelines.

### 2.5 Immune responses and antioxidant capacity

Serum samples were analyzed for key non-specific immune indices, namely lysozyme activity, total immunoglobulin (Total Ig), and alternative complement hemolytic activity (ACH50). Lysozyme activity was determined using the turbidimetric assay of Ellis (1990). Total Ig levels were measured following Siwicki and Anderson (1994), based on the variation in protein concentration pre and post precipitation with polyethylene glycol. ACH50 activity in serum was evaluated according to the method of Yano et al. (1992).

In addition, antioxidant status was assessed by measuring the concentrations of malondialdehyde (MDA), superoxide dismutase (SOD), and glutathione peroxidase (GPx), as well as catalase (CAT), in serum. These parameters were quantified using commercial kits (Zistchem Co., Tehran, Iran) following the protocols of Aebi (1984), McCord and Fridovich (1969), Paglia and Valentine (1967), and Ohkawa et al. (1979), respectively.

### 2.6 Stress factors

Cortisol in serum was evaluated using a standard commercial ELISA kit (Monobind Co., USA), based on a competitive sandwich ELISA procedure. Serum glucose was determined using commercially available diagnostic kits (Zistchem Co., Tehran, Iran).

#### 2.6.1 Heat stress

At the conclusion of the feeding trial, 10 *L. calcarifer* from each tank (i.e., 10 fish per replicate, 30 *L. calcarifer* per treatment) were transferred to special stress test aquaria. The water temperature was initially set at 23 °C and then raised by 3 °C per day using aquarium heaters until it reached 32 °C, where it was maintained for 3 days (Dawood et al., 2022). Following this thermal challenge, 3 *L. calcarifer* from each tank (i.e.,3 fish per replicate, 9 *L. calcarifer* per treatment) were randomly sampled. After blood collection and serum separation, glucose and cortisol were determined as physiological indicators of stress response.

### 2.7 Analysis

Prior to statistical evaluation, the dataset was assessed for distributional normality and equality of variances using the Shapiro–Wilk and Levene’s tests, respectively. All statistical procedures were conducted using SPSS software. Differences among dietary treatments were evaluated through one-way ANOVA, and significant variations were further examined using Tukey’s multiple comparison test to identify differences between individual groups. Statistical significance was determined at a probability level of *P* < 0.05. Data visualization and graphical representations were generated using GraphPad Prism. In addition, serum glucose and cortisol levels recorded before and after the temperature stress challenge were analyzed using two-way ANOVA, followed by suitable post hoc tests for multiple comparisons.

## 3 Results

### 3.1 Growth responses

The results indicated that FBW, WG (g), and SGR were significantly influenced by the experimental diets (Table 2). As the inclusion level of FWRP in the diets rose, FBW, WG, and SGR of the fish rose significantly relative to the control group. The greatest FBW (34.67), WG (28.79), and SGR (3.07) were recorded in the FWRP30 group, whereas the smallest values were observed in the control (FWRP0). Although SGR was notably higher in all FWRP-supplemented groups than in the control, no clear distinction (*P* = 0.005) was detected among the FWRP10, FWRP20, and FWRP30 treatments. Treatments also differed in FCR, with the most favorable FCR obtained in FWRP30 (*P* < 0.0001). Moreover, survival reached 100% across all groups, suggesting that dietary FWRP did not adversely affect fish health.

**Table 2:** Growth performance of *L. calcarifer* offered diets containing graded levels of FWRP over an 8-week feeding trial.

Treatments	IBW (g)^1^	FBW (g)^2^	WG (g)^3^	SGR (%/day)^4^	FCR^5^	Survival rate (%)^6^	Feed intake (g)
FWRP0	5.68	19.79^a^	14.11^a^	2.34^a^	1.63^a^	100	22.92
FWRP10	5.58	25.51^b^	19.94^b^	2.83^b^	1.06^a^	100	21.03
FWRP20	5.95	29.75^c^	23.81^c^	2.87^b^	0.78^b^	100	18.57
FWRP30	5.88	34.67^d^	28.79^d^	3.07^c^	0.70^c^	100	20.17
P- value	*P* = 0.130	*P* < 0.0001	*P* < 0.0001	*P* = 0.005	*P* < 0.0001	-	-
SEM	0.292	0.246	0.382	0.097	0.0315	-	-

1 IBW initial body weight.

2 FBW final body weight.

3 WG = FBW (g) − IBW (g).

4 SGR; %/day = 100 × [(Ln FBW − Ln IBW)/number of feeding days].

5 SR (%) = 100 × (final number of L. *calcarifer* /initial number of L. *calcarifer*).

6 FCR = FI (g)/WG (g) Columns with means marked by distinct letters highlight significant changes across treatments at *P* < 0.05 (mean ± pooled SEM, n = 25 fish per replicate; 75 fish per treatment).

### 3.2 Digestive enzyme profile in the intestine

The findings revealed that the activity of intestinal digestive enzymes of sea bass was notably affected (*P* < 0.05) among different FWRP levels (Table 3). The protease activity increased from 1.88 in the control group (FWRP0) to 2.52 in the FWRP30 treatment, and this difference was significant (*P* = 0.019). The amylase activity also rose notably (*P* < 0.0001) as FWRP levels increased in the diets, and the highest level was observed in the FWRP30 treatment (4.45), while the lowest level was detected in the control group (3.58). The lipase activity also significantly (*P* = 0.004) increased from 0.83 in the control group to 1.55 in the FWRP30 treatment. In general, increasing the level of FWRP in the diets improved the activity of digestive enzymes, and more lipase, amylase, and protease were observed in the FWRP30 treatment.

**Table 3:** Intestinal digestive enzymes of *L. calcarifer* offered diets containing graded levels of FWRP over an 8-week feeding trial.

Treatment/indices	Protease (U/mg protein)	Amylase (U/mg protein)	Lipase (U/mg protein)
FWRP0	1.88^a^	3.58^a^	0.83^a^
FWRP10	2.13^ab^	3.75^b^	1.11^ab^
FWRP20	2.23^ab^	4.19^c^	1.43^bc^
FWRP30	2.52^b^	4.45^d^	1.55^c^
P- value	*P* = 0.019	*P* < 0.0001	*P* = 0.004
SEM	0.106	0.027	0.102

Columns with means marked by distinct letters highlight significant changes across treatments at *P* < 0.05. Superscript letters indicate statistical groupings and do not represent the ranking of mean values (mean ± pooled SEM, n = 3 fish per replicate; 9 fish per treatment).

### 3.3 Liver enzymes

Hepatic enzyme activities in sea bass following 8 weeks of feeding with varying FWRP concentrations are presented in Table 4. ALP activity was significantly influenced by the dietary treatments (*P* < 0.001). The greatest activity was documented in the control group, whereas the smallest value was found in fish fed the FWRP30 diet. Also, the AST activity decreased (*P* < 0.001), and its lowest activity was observed in FWRP20 and FWRP30. In contrast, ALT activity did not differ appreciably among treatments (*P* = 0.070; Table 4).

**Table 4:** Hepatic enzyme activities of *L. calcarifer* offered diets containing graded levels of FWRP over an 8-week feeding trial.

Treatment/indices	ALP (U/L)	ALT (U/L)	AST (U/L)
FWRP0	44.10^a^	75.63	320.67^a^
FWRP10	41.36^a^	78.23	278.33^b^
FWRP20	29.23^b^	75.26	248.67^c^
FWRP30	25.86^c^	80.03	253.00^c^
P- value	*P* < 0.0001	*P* = 0.070	*P* < 0.0001
SEM	0.810	5.870	1.811

ALT: Alanine Aminotransferase, ALP: Alkaline Phosphatase, AST: Aspartate Aminotransferase. Columns with means marked by distinct letters highlight significant changes across treatments at *P* < 0.05. Superscript letters indicate statistical groupings and do not represent the ranking of mean values (mean ± pooled SEM, n = 3 fish per replicate; 9 fish per treatment).

### 3.4 Immune factors

**Fig. 1:**
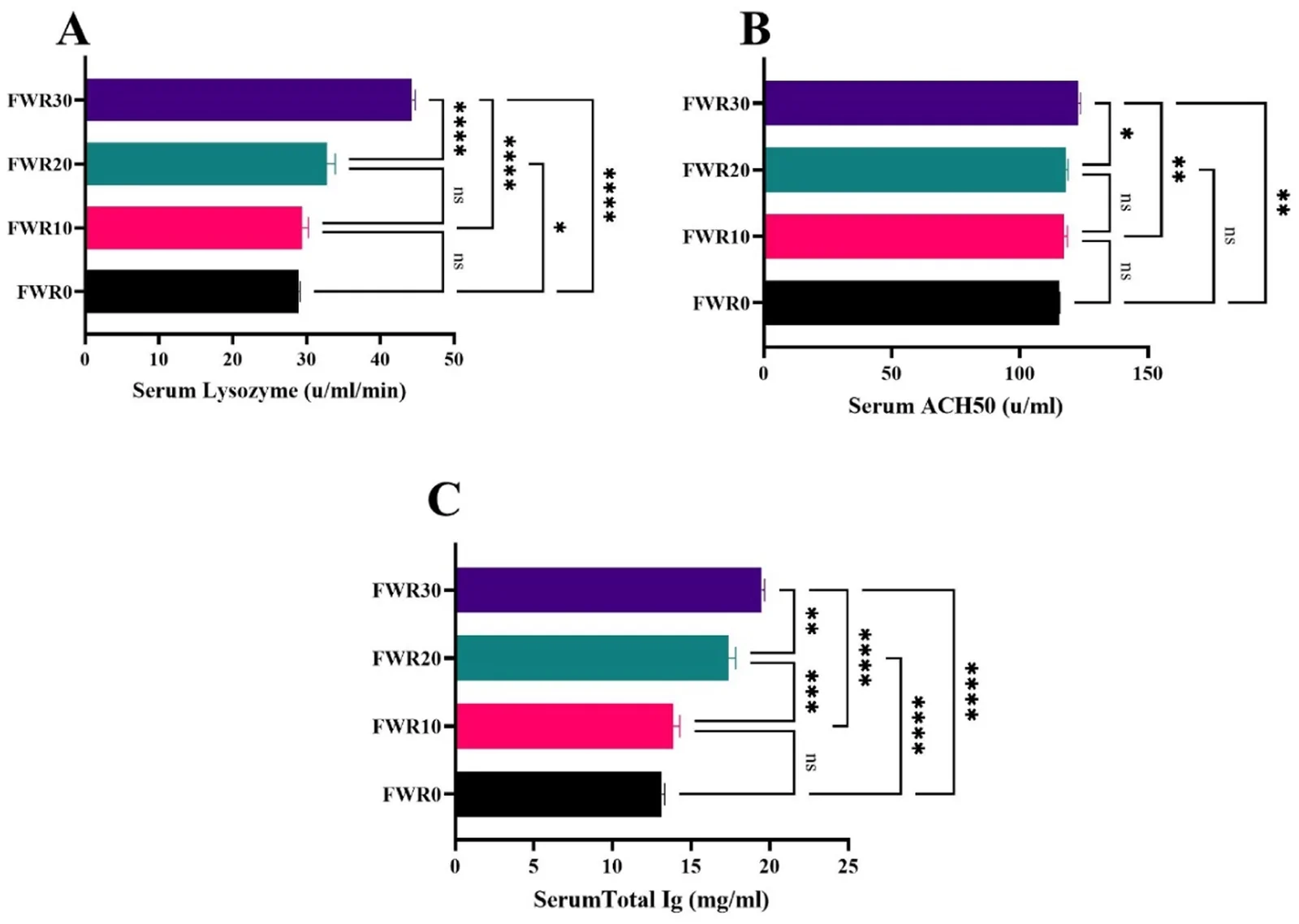
**A–C.** Immune parameters in *L. calcarifer* fed graded levels of fermented watermelon rind powder (FWRP) for 8 weeks. Bars labeled with varying letters denote significant changes across treatments (mean ± SEM, n = 3 fish per replicate; 9 fish per treatment). ns: *P* ≥ 0.05; *: *P* ≤ 0.05; **: *P* ≤ 0.01; ***: *P* ≤ 0.001; ****: *P* ≤ 0.0001.

Lysozyme activity (Fig. 1A), ACH50 (Fig. 1B), and total Ig (Fig. 1C) were influenced by dietary FWRP levels (*P* < 0.05). Lysozyme activity in the FWRP0 and FWRP10 groups was similar (*P* > 0.05), while the FWRP20 group showed a clear increase compared with both. The highest lysozyme activity was in the FWRP30 group, above all other groups (Fig. 1A). For ACH50, the FWRP0, FWRP10, and FWRP20 treatments had similar values (*P* > 0.05), but the FWRP30 treatment had the highest ACH50 activity (Fig. 1B). Total Ig concentration increased with FWRP level. FWRP0 and FWRP10 did not differ (*P* > 0.05), while fish fed the FWRP20 diet had higher total Ig than these two groups (Fig. 1C). The highest total Ig was in the FWRP30 group, greater than all other treatments (Fig. 1C).

### 3.5 Antioxidant

**Fig. 2:**
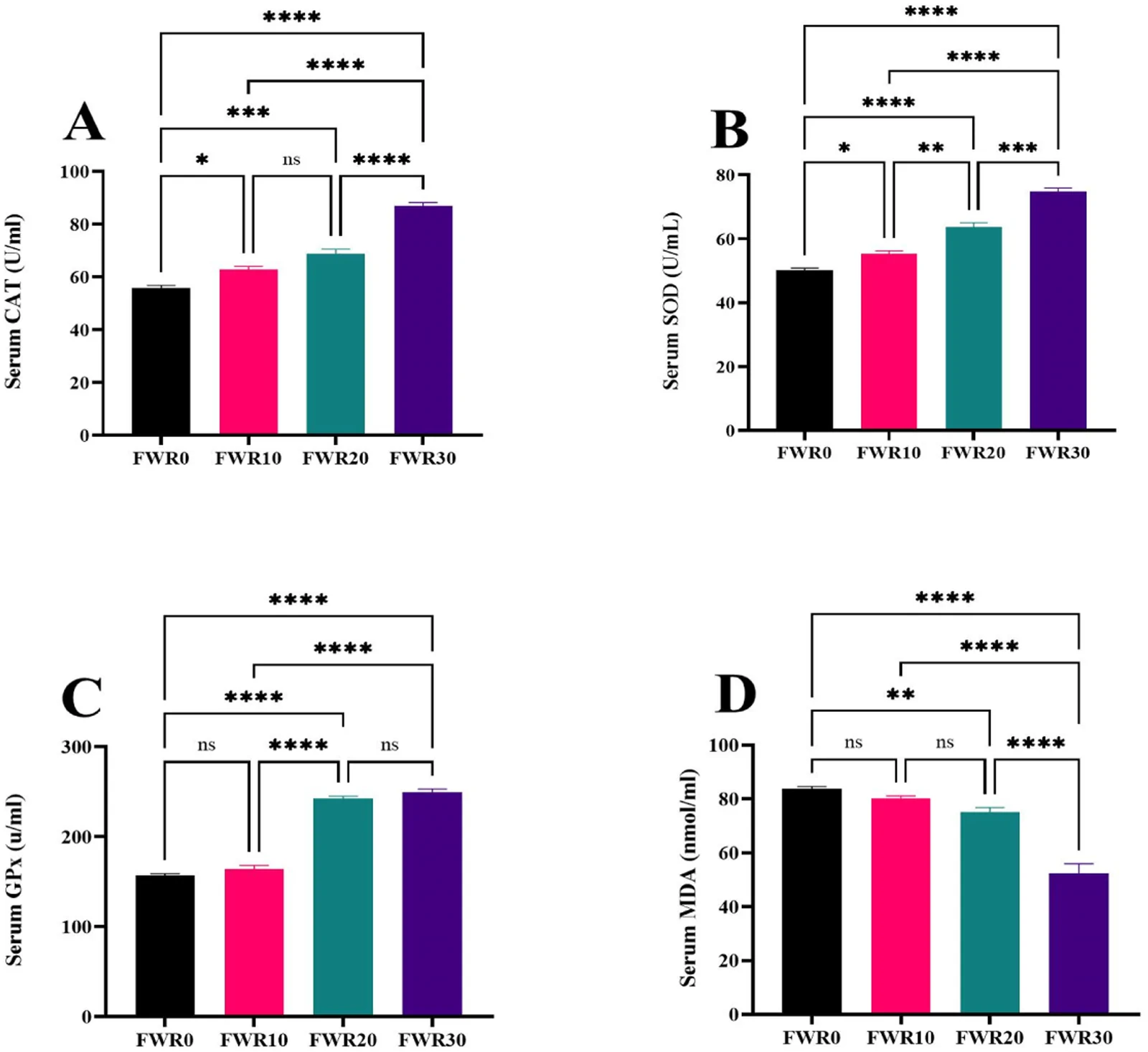
**A-D.** Antioxidant parameters in *L. calcarifer* fed graded levels of fermented watermelon rind powder (FWRP) for 8 weeks. Bars labeled with varying letters denote significant changes across treatments (mean ± SEM, n = 3 fish per replicate; 9 fish per treatment). ns: *P* ≥ 0.05; *: *P* ≤ 0.05; **: *P* ≤ 0.01; ***: *P* ≤ 0.001; ****: *P* ≤ 0.0001.

CAT, SOD, and GPx activities were significantly influenced by the dietary treatments (*P* < 0.05). CAT activity (Fig. 2A) rose progressively from FWRP0 to FWRP30, with each treatment differing notably from the others (*P* < 0.05); the highest activity was recorded in fish fed the FWRP30 diet. SOD activity (Fig. 2B) also increased in a dose-dependent manner as FWRP levels in the diet rose from FWRP0 to FWRP30. GPx activity (Fig. 2C) was substantially higher in the FWRP20 and FWRP30 groups than in FWRP0 and FWRP10 (*P* < 0.05), while FWRP0 and FWRP10 did not differ from each other (*P* > 0.05). MDA levels (Fig. 2D) declined as dietary FWRP increased. The lowest MDA concentration was noted in the FWRP30 group, and the highest in the control (FWRP0) group (*P* < 0.05).

### 3.6 Stress factors

**Fig. 3:**
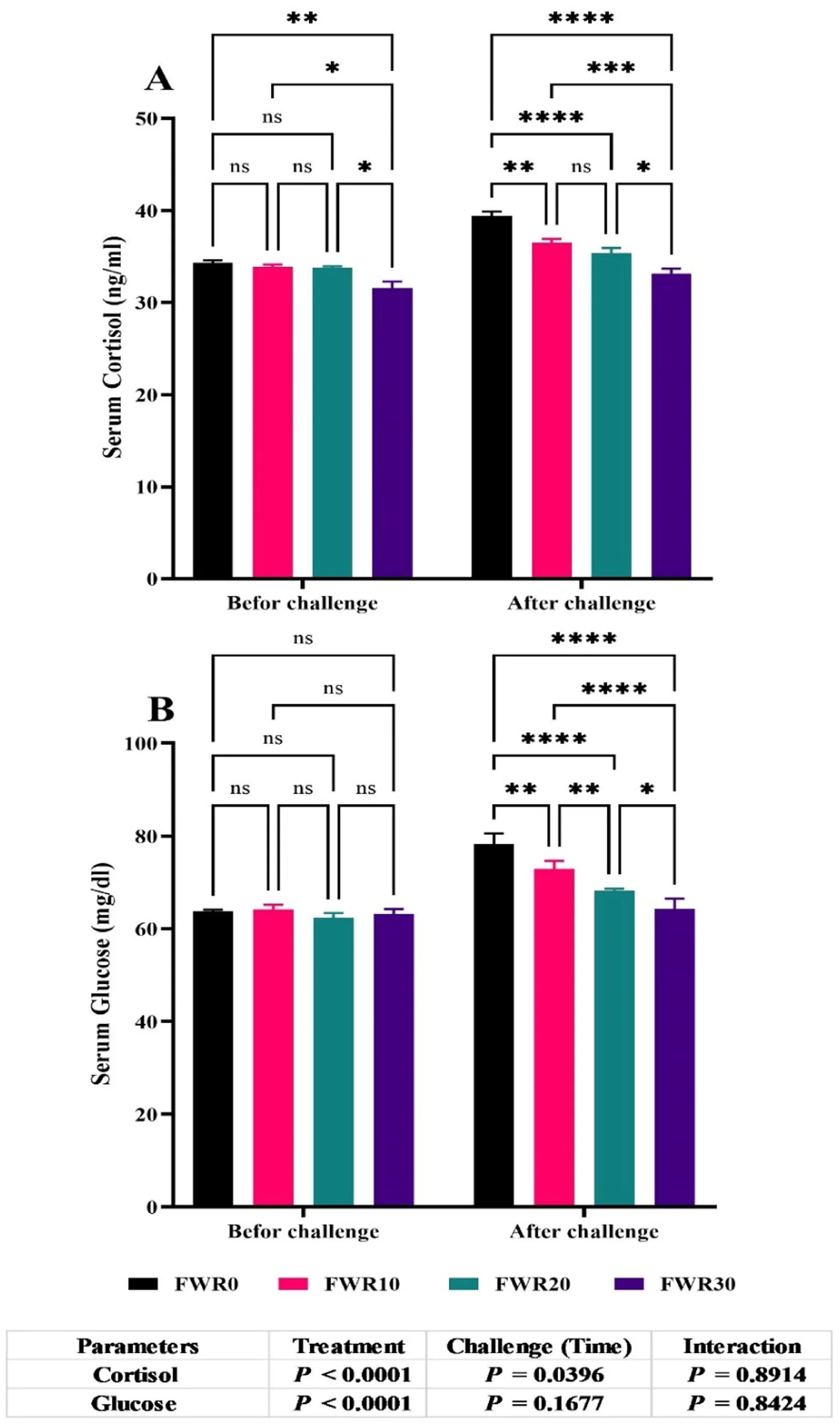
**A-B.** Stress factors in *L. calcarifer* fed graded levels of fermented watermelon rind powder (FWRP) for 8 weeks. Bars labeled with varying letters denote significant changes across treatments (mean ± SEM, n = 3 fish per replicate; 9 fish per treatment). ns: *P* ≥ 0.05; *: *P* ≤ 0.05; **: *P* ≤ 0.01; ***: *P* ≤ 0.001; ****: *P* ≤ 0.0001.

Serum cortisol showed a significant treatment effect in Asian sea bass, meaning the different diets caused clear differences among groups (*P* < 0.0001; Fig. 3A). The time effect was also significant, because cortisol increased significantly after challenge, indicating a stress response (*P* < 0.0396). However, the treatment × time interaction was not significant, so the pattern of cortisol change over time was similar across diets (*P* = 0.8914). Serum glucose also showed a significant treatment effect, which means the diets caused differences in glucose levels among the experimental groups (*P* < 0.0001; Fig. 3B). In contrast, the time effect was not significant, so glucose did not change significantly before and after challenge at the whole-group level (*P* = 0.1677). The treatment × time interaction was also not significant, showing that the glucose response over time was comparable among diets (*P* = 0.8424).

## 4 Discussion

In aquaculture research, a wide range of herbs and their derived products are routinely incorporated into fish feeds to improve nutrient utilization, promote growth, and enhance the survival of farmed aquatic species (Hoseinifar et al., 2020c; Lieke et al., 2020). In this study, juvenile *L. calcarifer* fed diets containing FWRP showed enhanced growth performance and feed efficiency, with the most pronounced improvements observed in the FWRP30 treatment. Our findings align with previous reports showing better growth in several fish species fed watermelon-based feed ingredients. Enhanced growth has been documented in African catfish (*Clarias gariepinus*) given diets containing *Citrullus lanatus* seed meal (Tiamiyu et al., 2015), Nile tilapia fed melon seed peel (Iheanacho et al., 2018), African catfish supplemented with watermelon syrup booster (Nwanevu et al., 2019), and Nile tilapia receiving watermelon rind powder (Van Doan et al., 2020, 2021). FWRP appears to be a nutritionally valuable ingredient with functional properties that can improve diet palatability, thereby stimulating feed intake. The observed increase in digestive enzyme activities may have contributed to more efficient digestion and nutrient utilization. In addition, the fiber and bioactive components present in FWRP may provide substrates that support beneficial intestinal microbial populations, which could further contribute to digestive processes and nutrient absorption. Including FWRP in Asian sea bass diets may therefore enhance intestinal digestive processes and nutrient uptake across the intestinal barrier (Van Doan et al., 2020, 2021). Collectively, these potential effects may improve feed utilization and nutrient assimilation, thereby contributing to the observed improvement in growth performance.

ALT and AST are key enzymes in protein metabolism, catalyzing the transfer of amino groups between α-keto acids and amino acids. In contrast, ALP is a hydrolase that cleaves phosphate groups from various molecules. These enzymes are commonly used as clinical biomarkers to assess hepatic and, in the case of ALP, bone-related physiological status. The current study showed that feeding Asian sea bass on FWRP diets led to marked declines in ALP and AST activities with no changes in ALT activities. The reductions in AST and ALP activities may suggest a favorable physiological response to FWRP supplementation and potentially improved hepatic status. Earlier work has shown that supplementing fish diets with various herbal extracts and phytochemicals can lower plasma ALP, ALT, and AST activities (Abdel-Tawwab et al., 2026; Hoseini et al., 2021; Yousefi et al., 2020, 2021). For example, increasing dietary ellagic acid reduced AST, ALT, and ALP levels, which was associated with improved liver-related physiological responses in yellow catfish (Wang et al., 2024). Similarly, dietary phytol concentrations significantly influenced plasma ALT, AST, and ALP activities in common carp (Hoseini et al., 2021).

In this study, adding WMRP to the diets of juvenile *L. calcarifer* led to clear increases in lysozyme activity, ACH50, and total Ig levels. Comparable improvements in immune parameters have been reported in other species: convict cichlid (*Amatitlania nigrofasciata*) fed polyphenol-rich agricultural byproducts (Hoseinifar et al., 2020a); common carp (*Cyprinus carpio* L.) receiving similar polyphenol sources; rainbow trout (*Oncorhynchus mykiss*) supplemented with olive (*Olea europaea* L.) waste (Hoseinifar et al., 2020b); common carp fed turmeric and white button mushroom powder (Giri et al., 2019; Hoseinifar et al., 2019a); and Nile tilapia given WMRP (Van Doan et al., 2020, 2021) or jackfruit waste (Abdel-Tawwab et al., 2025). Khanzadeh et al. (2026) further showed that *Sargassum ilicifolium* fermented with *L. plantarum* and *Saccharomyces cerevisiae* enhanced immune-related traits in *O. mykiss*, both before and after experimental challenge with *Streptococcus iniae*. Although no prior work has examined FWRP specifically in aquatic species, our results support its use as a functional feed additive for Asian sea bass. WRP is rich in β-carotene and vitamin C, compounds linked to local intestinal immunity and known to act as immunostimulants with antioxidant properties (Tarazona-Díaz et al., 2011). Additionally, it serves as a rich source of lycopene and the essential amino acid citrulline (Alagbe, 2018; Tarazona-Díaz et al., 2011). Citrulline, in particular, exhibits strong antioxidant potential due to its ability to neutralize hydroxyl radicals, thereby contributing to the reduction of oxidative stress (Ginguay et al., 2019).

The antioxidant defense system is commonly assessed through enzymatic indicators such as SOD, CAT, and GPx. Conversely, MDA, a product of ROS-induced lipid oxidation, serves as a marker of oxidative damage due to its ability to modify proteins and DNA, resulting in potential cellular toxicity (Lushchak, 2011). Together, MDA and the antioxidant enzymes SOD, CAT, and GPx serve as key indicators of health in aquatic animals exposed to different environmental and dietary conditions (Hoseini et al., 2021; Rajabiesterabadi et al., 2020). In Asian sea bass, dietary FWRP significantly affected SOD, CAT, and GPx activities as well as MDA levels. Fish fed FWRP-supplemented diets, especially at 30 g/kg feed, showed higher SOD, CAT, and GPx activities alongside a pronounced reduction in MDA. These effects likely reflect the high content of flavonoids and phenolic compounds in FWRP, which possess strong antioxidant properties (Khajeh et al., 2026). Previous research has similarly shown that feed additives can enhance antioxidant capacity in various aquatic species. For example, common carp fed diets containing 0.5% 1,8-cineole exhibited increased CAT, SOD, and GPx activities (Rajabiesterabadi et al., 2020). Dietary anthocyanins substantially raised SOD and CAT activities in the spleen and upregulated CAT, SOD, and GPx expression in the liver of Nile tilapia (Yilmaz, 2019). Abdel-Tawwab et al. (2026) also reported that Nile tilapia fed jackfruit waste–based diets showed improved antioxidant status through modulation of SOD, CAT, and GPx gene expression.

## 5 Conclusion

The results show that adding *A. niger*–fermented watermelon rind powder to the diet improves growth, digestive enzyme activities, antioxidant capacity, and immune function in *L. calcarifer*, especially under heat-stress conditions. The observed reductions in liver enzyme activities may further suggest a favorable physiological response to FWRP supplementation. Among the tested inclusion levels, 30 g/kg feed resulted in the greatest overall improvements in the measured responses; however, this level should not be considered a definitive optimal inclusion level, as higher inclusion levels were not evaluated in the present study. Overall, fermented watermelon rind appears to be a promising functional additive for enhancing fish health and resilience in intensive aquaculture. Nevertheless, the present study has some limitations, including the evaluation of a relatively limited range of FWRP inclusion levels and the absence of higher inclusion levels that could help determine the true optimal dose. In addition, the experimental duration and controlled experimental conditions may limit the direct extrapolation of these findings to long-term and commercial aquaculture production. Future studies should therefore investigate a broader range of FWRP inclusion levels, evaluate longer-term effects under commercial farming conditions, and investigate the molecular mechanisms involved, including gene expression related to antioxidant and immune pathways.

## Ethical approval

The study was performed under the approval and supervision of the University of Tehran Faculty of Sciences ethics and animal care committee (Approval No. 357, 8 November 2000).

## Ethics declaration

This study was conducted in accordance with the ARRIVE (Animal Research: Reporting of In Vivo Experiments) guidelines. This study was approved by The study was performed under the approval and supervision of the University of Tehran Faculty of Sciences ethics and animal care committee (Approval No. 357, 8 November 2000). (Approval No. Approval No. 357, 8 November 2000)

## Consent for publication

Not applicable.

## AI statement

Artificial intelligence methods were not utilized in this research.

## CRediT authorship contribution statement

**Ehsan Ahmadifar:** Writing – review & editing, Writing – original draft, Visualization, Validation, Supervision, Resources, Project administration, Methodology, Investigation, Conceptualization. **Ali Arshadi:** Writing – review & editing, Writing – original draft, Visualization, Validation, Methodology, Investigation, Conceptualization. **Sedigheh Mohammadzadeh:** Writing – review & editing, Writing – original draft, Visualization, Validation, Supervision, Investigation. **Majid Khanzadeh:** Writing – review & editing, Writing – original draft, Software, Methodology, Formal analysis. **Mostafa Khajeh:** Writing – review & editing, Writing – original draft, Visualization, Methodology, Conceptualization. **Amin Oujifard:** Writing – review & editing, Writing – original draft, Validation, Methodology, Investigation. **Mohsen Shahriari Moghadam:** Writing – review & editing, Writing – original draft, Visualization, Validation, Methodology, Investigation, Conceptualization. **Mohsen Abdel-Tawwab:** Writing – review & editing, Writing – original draft, Validation, Methodology, Investigation, Conceptualization.

## Declaration of competing interest

The authors declare that they have no known competing financial interests or personal relationships that could have appeared to influence the work reported in this paper.

## Data Availability

The data and supporting materials for this study are available from the corresponding author upon justified request.
